# PT-Level High-Sensitivity Magnetic Sensor with Amorphous Wire

**DOI:** 10.3390/s20010161

**Published:** 2019-12-26

**Authors:** Dongfeng He

**Affiliations:** National Institute for Materials Science, 1-2-1 Sengen, Tsukuba, Ibaraki 305-0047, Japan; he.dongfeng@nims.go.jp; Tel.: +81-29-859-2533

**Keywords:** magnetic sensor, FeCoSiB, amorphous wire, resonant circuit, eddy current testing

## Abstract

A picotesla (PT) level high-sensitivity magnetic sensor with amorphous wire was developed. The magnetic sensor was composed of a (Fe_0.06_Co_0.94_)_72.5_Si_2.5_B_15_ (FeCoSiB) amorphous wire with a coil wound around it. The amorphous wire had a diameter of 0.1 mm and a length of 5 mm. The coil was 30 turns. There was no electrical connection with the amorphous wire. The sensor was biased by an alternating current (AC) of about 1 MHz and a direct current (DC). To increase the sensitivity, a resonant circuit was used, and the signal amplitude of the magnetic sensor was increased 10 times from 10 mV/Gauss to about 100 mV/Gauss. The magnetic field resolution was improved 5 times from 30 pT/√Hz to 6 pT/√Hz. An eddy current testing system with a magnetic sensor was developed, and the artificial defects in an aluminum plate were evaluated.

## 1. Introduction

The magneto-impedance (MI) effect of amorphous wire made of soft ferromagnetic materials has been used to fabricate magnetic sensors with high sensitivities [1,2,3,4,5,6,7]. For these magnetic sensors, the amorphous wires were generally connected with an AC current source with the high frequency of over 10 MHz; and a detection coil was wound around the amorphous wire. The amplitude of the voltage generated at the detection coil corresponded to the external magnetic field. Mohri, K. et al. [8] and Dolabdjian, C. et al. [9] gave reviews about the high-sensitivity MI magnetic sensor. These MI magnetic sensors had high magnetic field resolution of PT level and were used to measure the biomagnetic fields [10,11] and magnetic nanoparticle detection [12,13,14]. Based on the off-diagonal giant magneto-impedance (GMI) effect in Co-rich glass-coated microwire, high-sensitivity GMI sensors were also developed [15,16]. The level of the equivalent magnetic noise of about 10 pT Hz/√Hz at a frequency of 300 Hz was obtained. Ma, J.J. and Uchiyama, T. [17] proposed a high-performance MI sensor system with a peak-to-peak voltage detector by synchronized switching, and the low-frequency noise was suppressed by using the MI gradiometer. Using the MI sensor, they successfully achieved a clear record of the spontaneous brain activity with SNR of about eight.

Some environmental parameters can dramatically affect the accuracy and reliability of the GMI sensor measurement, such as the temperature, the conductors nearby, or the stress on the amorphous wire. The effect of tensile stress on the amorphous wire GMI sensor has been investigated both experimentally and theoretically [18,19,20]. It was observed that the axial tensile stress induces the magneto-elastic anisotropy, which reduces the permeability of the amorphous wires. Tensile stress initially induced refined circumferential domain structure, which gives a large MI effect, but after a certain critical value, the larger stresses increase the magneto-elastic anisotropy that reduces the MI change. For a normal GMI sensor with electrical connection to the amorphous wire, both ends of the amorphous wire are soldered on substrates. The different thermal coefficient may influence the sensitivity and the noise performance of the GMI sensor.

He, D.F. et al. [21] developed high sensitivity magnetic sensor using (Fe_0.06_Co_0.94_)_72.5_Si_2.5_B_15_ (FeCoSiB) amorphous wire. For this magnetic sensor, there was no electrical connection with the wire. The AC bias current and the DC bias current were connected with a coil wound around the amorphous wire. Because there was no electrical connection with the amorphous wire and only one end of the wire was glued on a substrate, there was no stress effect for this magnetic sensor.

**Figure 1:**
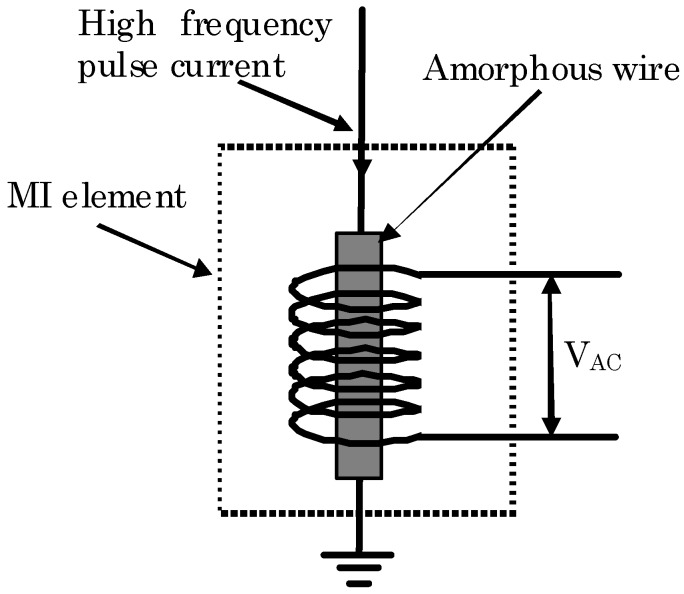
The magneto-impedance (MI) element composed of amorphous wire with an electrical connection between the amorphous and the AC high-frequency current.

Figure 1 shows the principle diagram of the normal MI magnetic sensor. For this kind of MI magnetic sensor, an electrical connection was needed, and a special method was proposed to make good electrical connection with the amorphous wire [22]. The electrical connection increased the complexity of element fabrication and caused the Joule heat loss of the material. Moreover, the MI elements with electrical connections were not suitable for some applications, such as a magnetic microscope, where the small distance of several micrometers between the sensor and the sample was needed. And the degradation of the electrical connection of the amorphous wire might also cause the failure operation of the sensor.

**Figure 2:**
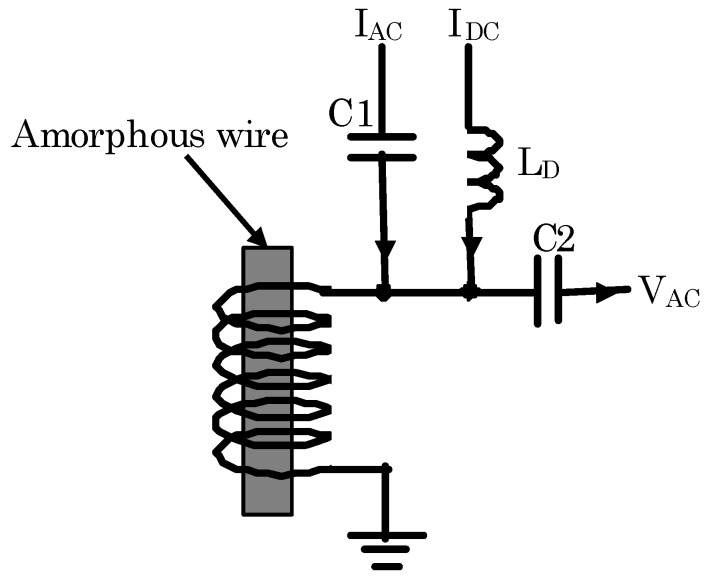
MI element composed of (Fe_0.06_Co_0.94_)_72.5_Si_2.5_B_15_ (FeCoSiB) amorphous wire without the electrical connection with the amorphous wire.

Figure 2 shows the configuration of another kind of magnetic sensor FeCoSiB amorphous wire [21]. There was no electrical connection with the amorphous wire. The magnetic field resolution of about 30 pT/√Hz was obtained. Both AC current and DC current were used to bias the magnetic sensor. The DC current was used to produce a DC magnetic field to make the amorphous wire near to the saturation of the magnetization. Due to the nonlinearity of the B-H curve near the saturation, the permeability of the amorphous wire changed with the external magnetic field. That caused the amplitude change of the AC voltage across the coil. This is the principle of this kind magnetic sensor [21]. Because there was no electrical connection with the amorphous wire, it would be convenient to construct application systems using this kind of magnetic sensor.

The signal amplitude of the magnetic sensor was about 10 mV/Gauss. If the equivalent input voltage noise spectrum of the preamplifier is Vin, and the signal amplitude of the magnetic sensor is δV/δB, the magnetic field resolution of the magnetic sensor Bn can be estimated by the equation:

Bn = Vin/(δV/δB).(1)

AD829 was used to make the preamplifier. The equivalent input voltage noise Vin was about 2 nV/√Hz when the frequency was over 100 Hz. δV/δB was about 10 mV/Gauss. The magnetic field resolution Bn was estimated to be about 20 pT/√Hz. Based on Equation (1), there are two ways to improve the magnetic field resolution: One way is to reduce the noise of the preamplifier, and another way is to increase the signal amplitude (δV/δB) of the magnetic sensor.

In this paper, a resonant circuit was used to increase the signal amplitude δV/δB and the magnetic field resolution of the magnetic sensor was improved.

## 2. PT-level High Sensitivity Magnetic Sensor with Resonant Circuit

**Figure 3:**
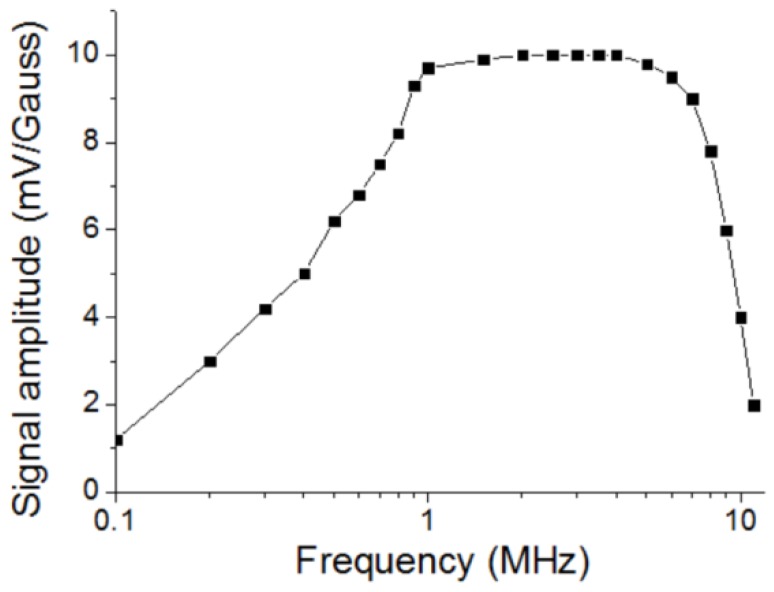
Signal amplitude of the magnetic sensor changing with the frequency of the AC bias current.

To determine the frequency of the bias AC current, the signal amplitude change with the frequency was measured. Figure 3 shows the result. The signal amplitude increased with the frequency when the frequency was below 1 MHz; the signal kept constant when the frequency was between 1 MHz and 7 MHz, and the signal decreased with the frequency when the frequency was over 7 MHz. Considering that it would be easier to design the circuit with lower frequency, 1 MHz was chosen for the frequency of the AC bias current.

**Figure 4:**
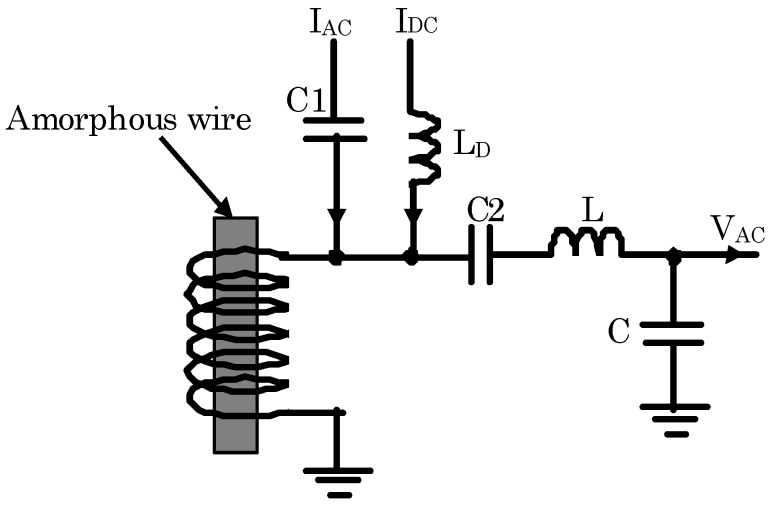
Schematic diagram of the amorphous wire magnetic sensor with the inductor-capacitor (LC) resonant circuit. The resonant frequency was the same as the frequency of the AC bias current.

**Figure 5:**
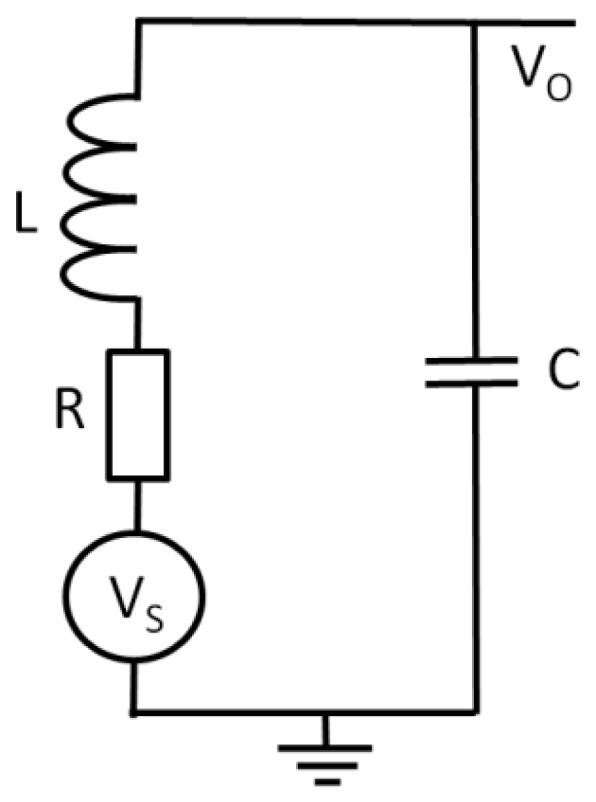
Equivalent circuit of the resonant circuit.

To increase the signal amplitude, a resonant circuit was used between the sensor element and the preamplifier. Figure 4 shows the schematic diagram of the magnetic sensor with the resonant circuit. A FeCoSiB amorphous wire with a diameter of 0.1 mm and length of 5 mm was used, and a 30 turn coil made of 0.1 mm copper wire was wound around the amorphous wire. AC current and DC current were used to bias the sensor. The capacitors C1, C2, and an inductor L_D_ were used to isolate the AC bias current, the DC bias current, and the signal across the coil. The resonant circuit was composed of an inductor L and a capacitor C. The values of C1 and C2 were much bigger than the value of the capacitor C. The resonant frequency of the resonant circuit was adjusted to be the same as the frequency of the AC bias current. Figure 5 shows the equivalent circuit of the resonant circuit. The equivalent resistance of the resonant circuit was R. The resonant frequency f was determined by:

(2) $$f=\frac{1}{2\pi \sqrt{\mathrm{LC}}}.$$

The quality factor Q of the resonant circuit was

(3) $$Q=\frac{1}{2\pi \mathrm{fCR}}.$$

For a voltage source V_S_, the current flow in the resonant circuit at the resonant frequency was I = V_S_/R. Then the voltage V_O_ across capacitor C was

(4) $$V_{O}=\frac{I}{2\pi \mathrm{fC}}=\frac{V_{S}}{2\pi \mathrm{fCR}}={\mathrm{QV}}_{S}$$

The Q value of the coil made of copper wire was about 10. Therefore, the voltage amplitude could be amplified by about 10 times using the resonant circuit.

The frequency of the AC bias current was 1 MHz, and the amplitude of the AC current was about 10 mA. The DC bias current was about 50 mA. The inductor L and the capacitor C of the resonant circuit were 22 μH and 1 nF respectively; and the resonant frequency of the resonant circuit was near to 1 MHz.

**Figure 6:**
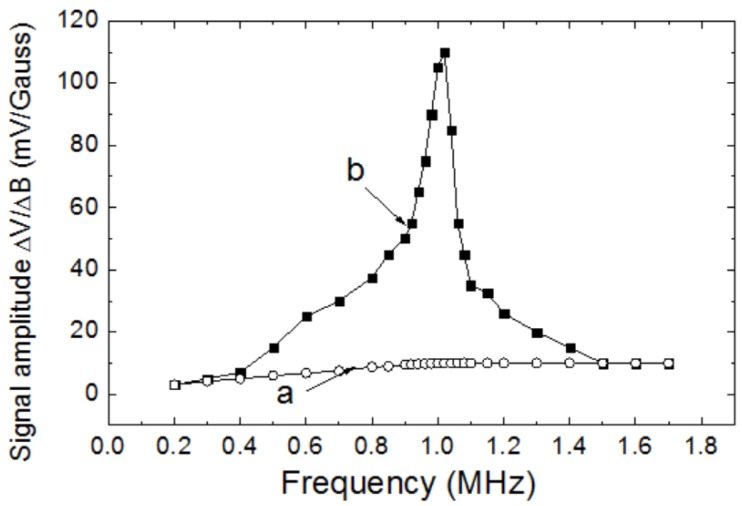
Signal amplitudes of the FeCoSiB amorphous wire magnetic sensor at different frequencies. a: the magnetic sensor without the resonant circuit. b: the magnetic sensor with the resonant circuit.

The signal amplitudes of the magnetic sensor with and without the resonant circuit were measured. Figure 6 shows the results. The line “a” in Figure 6 shows the signal amplitudes ΔV/ΔB of the magnetic sensor without the resonant circuit, and the line “b” in Figure 6 shows the signal amplitude ΔV/ΔB of the magnetic sensor with the resonant circuit. At the resonant frequency of about 1 MHz, the signal amplitude was increased by about 10 times: from about 10 mV/Gauss to about 100 mV/Gauss. Using Equation (1), the magnetic field resolution was estimated to be about 2 pT/√Hz when the resonant circuit was used. This was a theoretical value. For a real magnetic sensor, the magnetic field resolution of 6 pT/√Hz was achieved.

**Figure 7:**
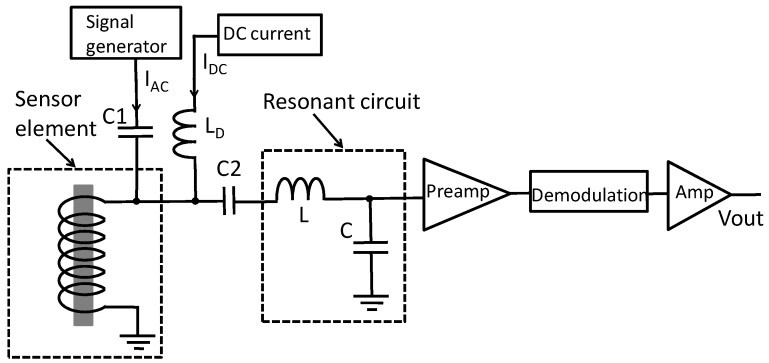
Block diagram of the driving circuit for the FeCoSiB amorphous wire magnetic sensor with the resonant circuit. The resonant circuit was composed of an inductor L and a capacitor C, which was used to increase the signal amplitude of the magnetic sensor.

Figure 7 shows the block diagram of the driving circuit for the magnetic sensor with the resonant circuit. The sensor was biased by an AC current and a DC current. The AC current and the DC current were connected with the coil wound around the amorphous wire. There was no electrical connection with the amorphous wire. The frequency of the AC current was 1 MHz. The 1 MHz bias current and the 1 MHz signal from the sensor could pass through the capacitor C1 and C2. The DC current I_DC_ could pass through the inductor L_D_. The inductor L and the capacitor C composed a resonant circuit with the resonant frequency of about 1 MHz, which was same as the frequency of the AC bias current. The AC voltage after the resonant circuit was amplified by the preamplifier. AD829 was used to make the preamplifier with the gain of 20 dB. A demodulator made by a diode was used to get the amplitude change of the AC signal. The output voltage of V_OUT_ was used to measure the applied magnetic field. The DC voltage of the output can be adjusted by a potentiometer. It was adjusted to zero when the applied magnetic field was zero.

**Figure 8:**
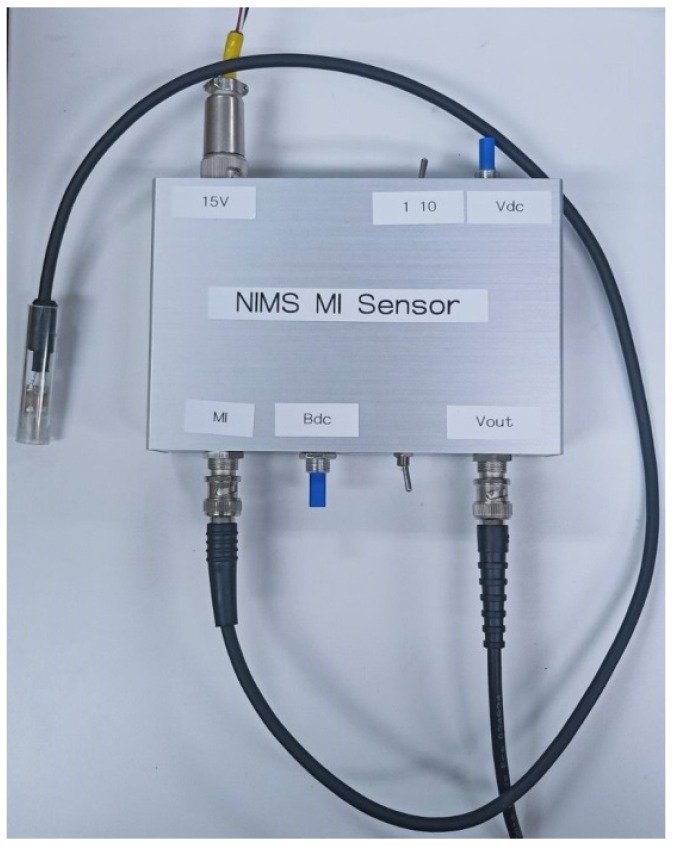
FeCoSiB amorphous wire magnetic sensor and the driving circuit.

**Figure 9:**
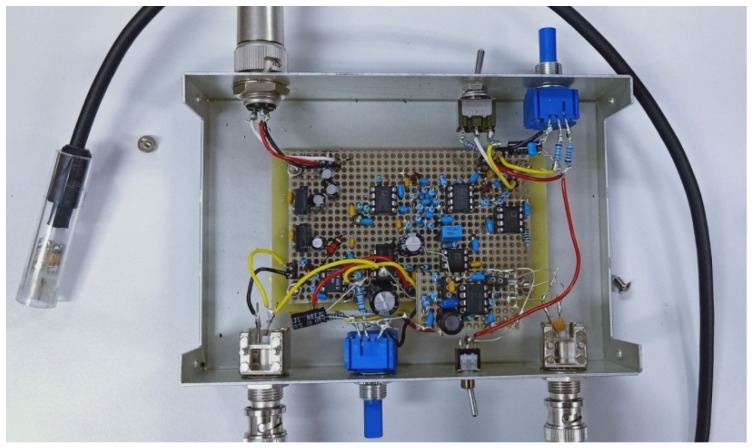
Circuit board of the driving circuit for the FeCoSiB amorphous wire magnetic sensor.

The driving circuit was hand made. Figure 8 and Figure 9 show the images of the driving circuit. Only one coaxial cable was used to connect the sensor element with the driving circuit. Thus, the sensing part of the magnetic sensor can be made very small for some special application where only a small space is allowed. The potentiometer Bdc in the driving circuit was used to adjust the DC bias current, and the potentiometer Vdc was used to adjust the DC level of the output voltage.

**Figure 10:**
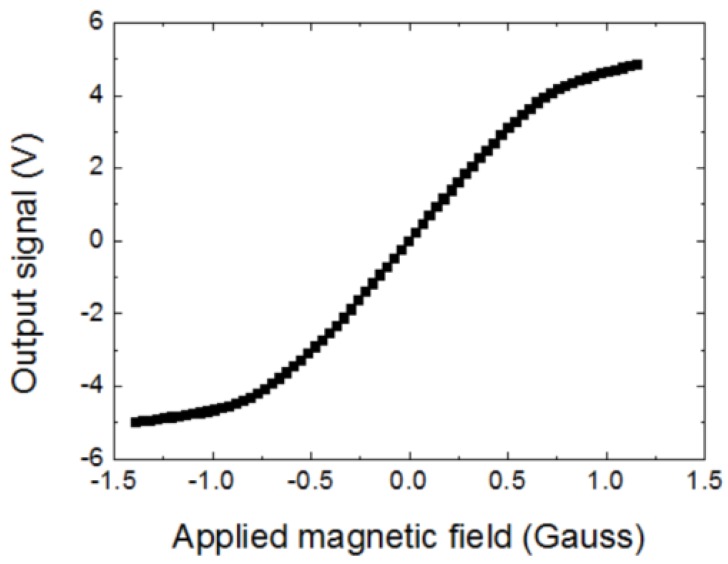
Magnetic response of the FeCoSiB amorphous wire magnetic sensor.

The magnetic sensor was put in a Helmholtz coil to measure the magnetic field response of the magnetic sensor. Figure 10 shows the response of the magnetic sensor to the applied magnetic field. The response was nearly linear when the applied magnetic field was in the range of −0.7 Gauss to +0.7 Gauss. In the linear response range, the voltage/magnetic field transfer factor ΔV/ΔB of the magnetic sensor was about 5 V/Gauss. The earth magnetic field is about 0.5 Gauss. Therefore, there is no difficulty for the magnetic sensor to operate in the earth’s magnetic field.

Using a spectrum analyzer of HP89441A, the voltage noise spectrum of the output signal of the magnetic sensor Vn was measured. From Figure 7, ΔV/ΔB was about 5 V/Gauss. The magnetic field noise spectrum Bn was calculated by Equation (5).

(5) $$\mathrm{Bn}=\frac{\mathrm{Vn}}{\Delta V/\Delta B}.$$

**Figure 11:**
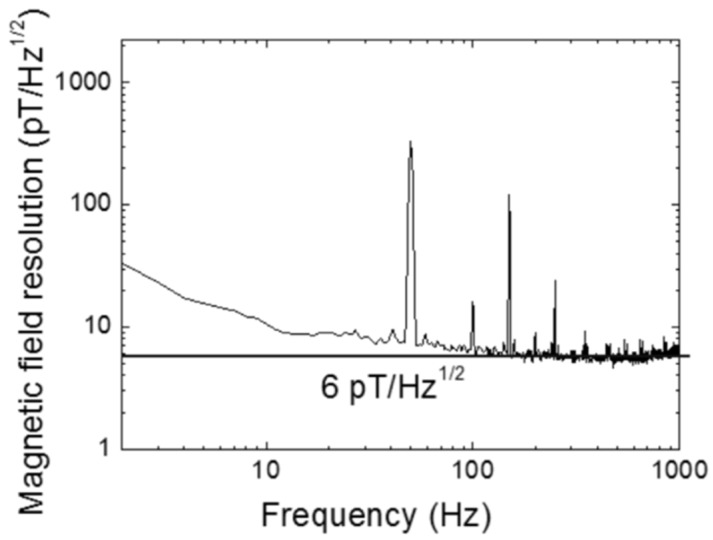
Magnetic field noise spectrum of the FeCoSiB amorphous wire magnetic sensor measured in a permalloy shielding box.

Figure 11 shows the magnetic field noise spectrum of the magnetic sensor with the resonant circuit. The noise spectrum was measured in a three-layer permalloy shielding box. The peaks on the spectrum were the 50 Hz line inference and its harmonics. The magnetic field noise spectrum was also called the magnetic field resolution. The magnetic field resolution was about 6 pT/√Hz at the frequencies over 200 Hz and it was about 10 pT/√Hz at about 10 Hz.

**Figure 12:**
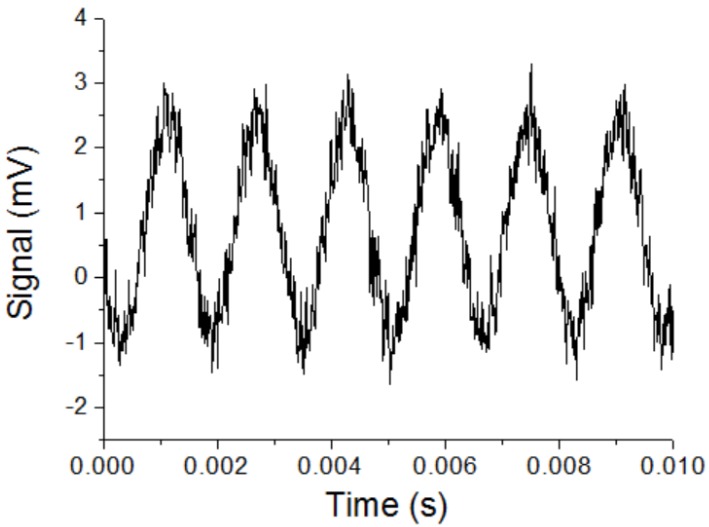
Output signal of the magnetic sensor for the applied magnetic field with a frequency of 630 Hz and the amplitude of 400 pT. A band-pass filter with a bandwidth of 100 Hz was used.

To check the magnetic field resolution of the magnetic sensor, the sensor was put in a Helmholtz coil, and a magnetic field with the frequency of 630 Hz and the amplitude of about 400 pT was applied to the sensor. An amplifier with the gain of 200 times and a band-pass filter with the center frequency of 630 Hz and the bandwidth of 100 Hz were used after the output of the magnetic sensor. Figure 12 shows the output signal of the magnetic sensor. The signal amplitude ΔV was 4 mV for the applied magnetic field ΔB = 400 pT. The noise level of the output signal Vnoise was about 0.7 mV. The bandwidth Δk was 100 Hz. The experimental magnetic field resolution could be estimated by Equation (6). It was about 7 pT/√Hz, which was similar to the results measured by the spectrum analyzer.

(6) $$\mathrm{Bn}=\frac{\mathrm{Vn}\cdot \Delta B}{\Delta V\cdot \sqrt{\Delta k}}$$

**Figure 13:**
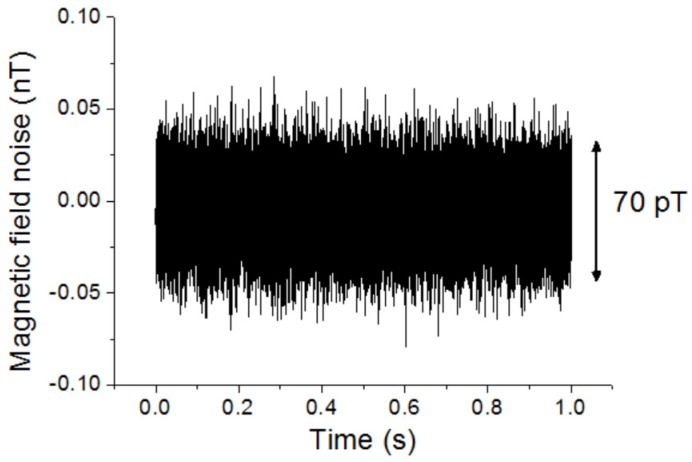
The magnetic field noise of the magnetic sensor at 630 Hz with the observation bandwidth of about 100 Hz.

**Figure 14:**
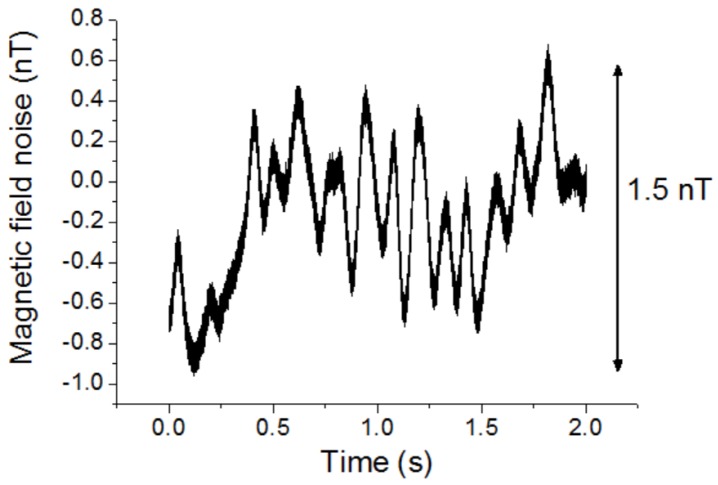
DC magnetic field noise of the magnetic sensor with a 10 Hz low-pass filter.

The magnetic field noise of the magnetic sensor at a higher frequency of 630 Hz and the DC magnetic field noise were measured. To reduce the influence of environmental noise, the magnetic sensor was put in a magnetic shielding box. Figure 13 shows the magnetic field noise of the magnetic sensor at 630 Hz. A band-pass filter with the center frequency of 630 Hz and the bandwidth of 100 Hz were used. The magnetic field noise was about 70 pT with the observation bandwidth of 100 Hz. The noise was similar when the frequency was over 100 Hz. Figure 14 shows the DC magnetic field noise with a 10 Hz low-pass filter. It was about 1.5 nT.

**Figure 15:**
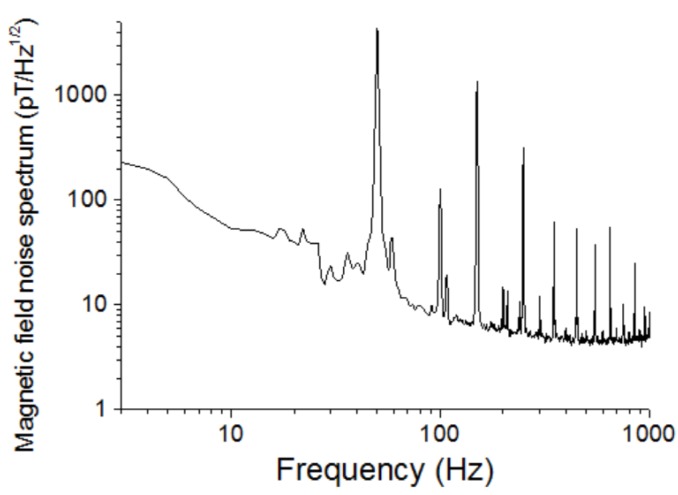
Magnetic field noise spectrum measured by the FeCoSiB amorphous wire magnetic sensor in the laboratory without shielding.

The magnetic field noise spectrum was also measured in our laboratory without shielding. Figure 15 shows the results. The amplitude of the 50 Hz line interference was about 4 nT, and the low-frequency noises were also increased. 

## 3. Eddy Current Testing Using the FeCoSiB Amorphous Magnetic Sensor

**Figure 16:**
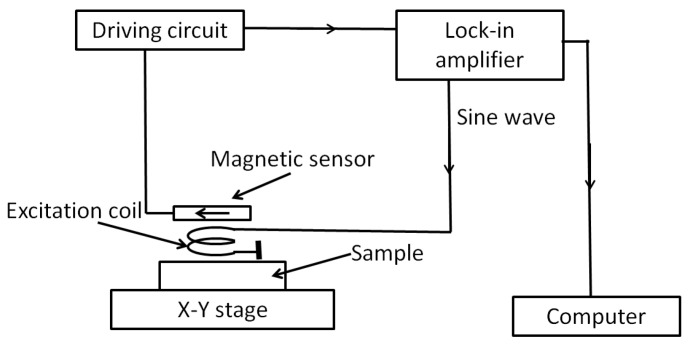
Block diagram of the eddy current testing (ECT) system with the FeCoSiB amorphous wire magnetic sensor.

Eddy current testing (ECT) is an effective way to evaluate the defects in conductive materials. For normal ECT equipment, an inductive coil is used, and mainly surface defects are detected. To detect a deep defect, magnetic sensors with high sensitivity at low frequency are used [23,24,25]. The ECT system using the FeCoSiB amorphous magnetic sensor was developed [26]. Figure 16 shows the setup of the ECT system.

The sine wave signal produced by the lock-in amplifier was sent to the excitation coil to produce an AC magnetic field. Then, eddy current was induced in the sample. The magnetic sensor was used to detect the magnetic field produced by the eddy current. The output signal of the magnetic sensor was sent to the lock-in amplifier. The outputs of the lock-in amplifier were sent to a computer. If there were some defects in the sample, the eddy current changed, then the outputs of the lock-in amplifier changed, and the defects could be detected. This is the principle of the ECT method. In our experiments, the excitation coil was 20 turns with a diameter of approximately 3 mm. The amplitude of the AC current flow in the excitation coil was about 20 mA, and the frequency was 1 kHz. The magnetic field produced by the excitation coil was along the vertical direction, and the sensing direction of the magnetic sensor was along the horizontal direction. The influence of the background magnetic field produced by the excitation coil was canceled by this configuration. The sample was put on an X-Y stage for the scanning.

**Figure 17:**
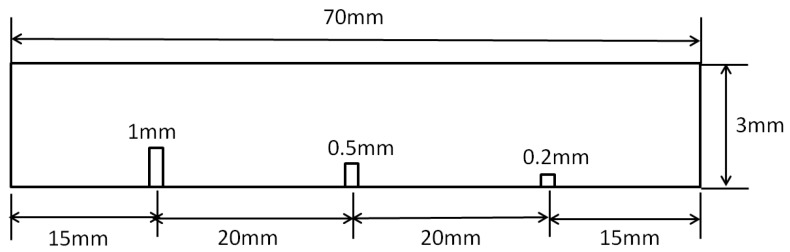
The sample of the aluminum plate with three artificial slits with a width of 0.2 mm and depths of 1 mm, 0.5 mm, and 0.2 mm.

Figure 17 shows the sample of an aluminum plate. The dimensions of the plate were 7 × 3 cm^2^. The thickness of the aluminum plate was 3 mm. Three slits with the same width of 0.2 mm and different depths of 1 mm, 0.5 mm, and 0.2 mm were made in the aluminum plate. The distance between the slits was about 2 cm.

For the ECT method, the detection depth is determined by the penetration depth of the eddy current. The penetration depth can be estimated by the following formula:

(7) $$\delta =\frac{I}{\sqrt{\pi f\mu \sigma }}$$

δ is the penetration depth of the eddy current, μ is the magnetic permeability, σ is the electrical conductivity of the material, and f is the frequency of the excitation magnetic field. For the aluminum plate, the electrical conductivity σ was about 3.8 × 10^7^ S/m, the magnetic permeability μ = μ_0_ = 4π × 10^−7^ H/m. Using Equation (7), we can calculate the penetration depth. It was about 2.6 mm at a frequency of 1 kHz. To detect the defects with the depths with the depth of 2 mm, 2.5 mm, and 2.8 mm in the aluminum plate, we chose the excitation frequency of about 1 kHz.

**Figure 18:**
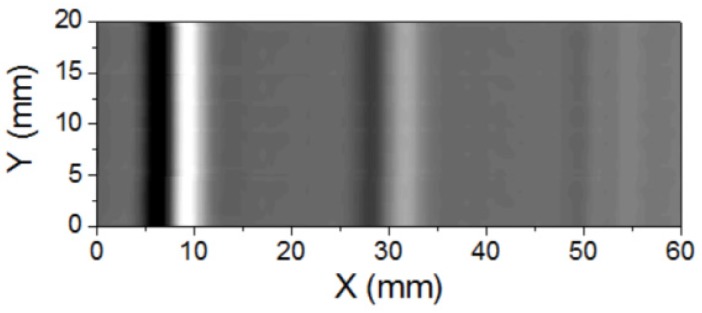
2D graph of the scanning results.

Figure 18 shows the scanning result using the ECT system. The scanning was done on the back side, and the scanning speed was about 1 cm/s. The amplitude output signal of the lock-in amplifier was used. The scanning area was 20 × 60 mm^2^. The artificial slits with the depths of 1 mm and 0.5 mm can be clearly observed. The signal produced by the slit with the depth of 0.2 mm was small.

## 4. Conclusions

Using an LC resonant circuit, the signal of the FeCoSiB amorphous wire magnetic sensor was improved 10 times from 10 mV/Gauss to 100 mV/Gauss, and the magnetic field resolution of the magnetic sensor was improved from 30 pT/√Hz to 6 pT/√Hz. The magnetic field noise at 630 Hz was about 70 pT when a band-pass filter with the bandwidth of 100 Hz was used. The DC magnetic field noise was about 1.5 nT when a 10 Hz low-pass filter was used. Using the magnetic sensor, the eddy current testing system was constructed, and the artificial defects in an aluminum plate were successfully detected.
